# Recent progress in the use of ‘omics technologies in brassicaceous vegetables

**DOI:** 10.3389/fpls.2015.00244

**Published:** 2015-04-14

**Authors:** Katja Witzel, Susanne Neugart, Silke Ruppel, Monika Schreiner, Melanie Wiesner, Susanne Baldermann

**Affiliations:** ^1^Leibniz-Institute of Vegetable and Ornamental Crops Großbeeren/Erfurt e.V.Großbeeren, Germany; ^2^Institute of Nutritional Science, University of PotsdamNuthetal, Germany

**Keywords:** genomics, transcriptomics, metabolomics, proteomics, crop, microbiomics

## Abstract

Continuing advances in ‘omics methodologies and instrumentation is enhancing the understanding of how plants cope with the dynamic nature of their growing environment. ‘Omics platforms have been only recently extended to cover horticultural crop species. Many of the most widely cultivated vegetable crops belong to the genus *Brassica*: these include plants grown for their root (turnip, rutabaga/swede), their swollen stem base (kohlrabi), their leaves (cabbage, kale, pak choi) and their inflorescence (cauliflower, broccoli). Characterization at the genome, transcript, protein and metabolite levels has illustrated the complexity of the cellular response to a whole series of environmental stresses, including nutrient deficiency, pathogen attack, heavy metal toxicity, cold acclimation, and excessive and sub-optimal irradiation. This review covers recent applications of ‘omics technologies to the brassicaceous vegetables, and discusses future scenarios in achieving improvements in crop end-use quality.

## Introduction

Brassicaceous vegetables, which are cultivated worldwide, belong to the taxa *Brassica oleracea* (cabbage, broccoli, cauliflower, kale, Brussels sprouts, collard greens, savoy, kohlrabi, and Chinese kale) and *B. rapa* (turnip, mizuna, napa cabbage, cime di rapa, and turnip rape). Just as for crops generally, maintaining their productivity in the light of incipient climate change and the dynamically changing pest and pathogen community represents a major challenge for the biotechnologist and the plant breeder (Augustine et al., 2014). The importance of this class of vegetables lies not only in their contribution to the vitamin and mineral components of the human diet, but also in their beneficial effect on human health, which reflects the action of the glucosinolates, a group of secondary plant metabolites almost exclusively associated with this plant family (Higdon et al., 2007; Jeffery and Araya, 2009; Wu et al., 2013). The aliphatic glucosinolates (and their break down products) have attracted scientific attention (Reichelt et al., 2002; Halkier and Gershenzon, 2006; Sonderby et al., 2010; Wittstock and Burow, 2010); of particular note in this context is the anti-carcinogen isothiocyanate sulforaphane, the major break down product of glucoraphanin in broccoli (Fahey et al., 1997; Shapiro et al., 2001, 2006).

**FIGURE 1:**
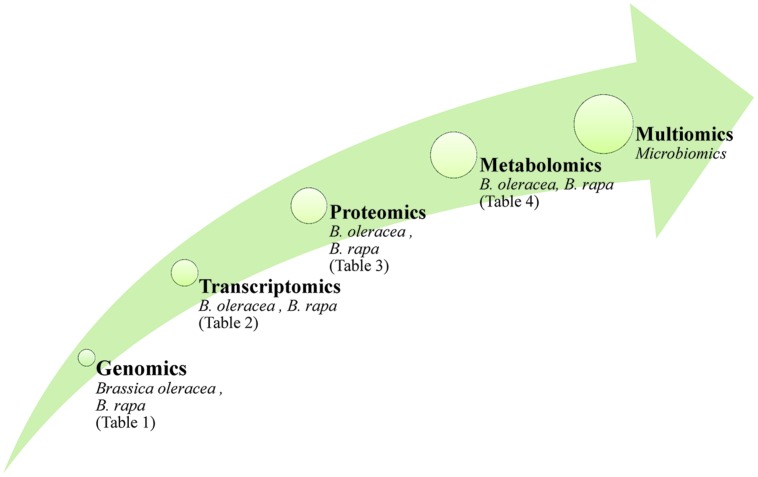
**The application of ‘omics technologies in brassicaceous vegetables.** Arrow size reflect the complexity of the molecular data and the impact on phenotype.

The use of ‘omics technologies, which current gather information either at the DNA, RNA, protein or metabolite levels, can potentially provide a comprehensive picture of cellular physiology. DNA sequence (although not the epigenome) is largely independent of the growing environment, while the transcriptome, proteome and metabolome are all highly responsive (**Figure 1**). Tailoring a crop cultivar to a specific environment is the central challenge for the plant breeder, and reflects the reality that genotype on its own will not generally be sufficient to support a biotechnology-driven crop improvement program. Rather, a combination of one or more of the ‘omics platforms is required to deliver reliable information. Such multiple ‘omics data sets tend to be very large, as they represent a series of time point and/or treatment samplings; their analysis can only be addressed computationally (Bieda, 2012; Kohl et al., 2014; Schumacher et al., 2014). The demand for better data processing led to the release of new software packages freely available or provided by vendors (examples are given in Katajamaa and Oresic, 2007), however, the visualization of multi-omic data sets remains an important task for bioinformatics. Practical tools should create clear and meaningful visualizations without being overwhelmed by the complexity of the data sets (recent approaches are discussed by Gehlenborg et al., 2010).

The prior acquisition of a full genome sequence aids materially in the interpretation of such data. The genome sequence of the model plant *Arabidopsis thaliana*, a member of the *Brassicaceae* family, has been known for nearly 15 years (Kaul et al., 2000), and the full genome sequences of both *B. rapa* (Wang et al., 2011) and *B. oleracea* (Ayele et al., 2005; Liu et al., 2014b) have been published more recently. Substantial amounts of transcript-based data have been acquired for *B. oleracea* (Gao et al., 2014; Izzah et al., 2014; Kim et al., 2014).

## Genomics

Genomic research has a great capability in speeding up breeding processes and several applications for crop improvement, through, e.g., marker-assisted selection and gene pyramiding. In case of brassicaceous vegetables, several populations have been generated to establish linkage maps using simple sequence repeat (SSR), amplified fragment length polymorphism (AFLP), nucleotide binding site or expressed sequence tag (EST) markers with the aim to genetically localize favorable traits by quantitative trait locus (QTL) analysis (summarized in **Table 1**). A survey of available online tools, covering mapping populations, linkage maps, gene sequences, and QTL, is presented by Li et al. (2013). Linkage maps were generated by crossing different genotypes of *B. oleracea* (Camargo et al., 1997; Iniguez-Luy et al., 2008) and *B. oleracea* var. *botrytis* (Gu et al., 2008). In the previous examples, a segregating offspring population was used to establish marker order and spacing. The same was shown for doubled haploid lines where offspring are homozygous (see Pink et al., 2008 for review). A doubled haploid population derived from crosses of two broccoli cultivars was genotyped by SSR and AFLP markers and QTL analysis identified loci for horticulturally important characteristics (Walley et al., 2012). Plant genetic resources have been characterized using AFLP markers to assess the huge genetic diversity present in gene banks, including Dutch and Italian *B. oleracea* (van Hintum et al., 2007; Maggioni et al., 2014) and Czech *B. oleracea* var. *capitata* accessions (Faltusova et al., 2011). A *B. rapa* collection consisting of 239 accessions was genotyped using SSR markers and subsequent association mapping identified two markers associated with flowering time (Zhao et al., 2010). Genetic basis of flowering time was also investigated in *B. rapa* by association mapping using natural variation and recombinant inbred lines (Lou et al., 2011). Phylogenetic relationships were established in *B. rapa* subspecies by AFLP markers (Takuno et al., 2007). The development of genetic maps from *Brassica* vegetables paved the way for QTL analysis and, together with accurate phenotyping, morphological, qualitative and yield traits were evaluated in different species. QTL mapping was conducted for eleven yield- and heading-related traits in doubled haploid Chinese cabbage lines and identified 46 main QTL (Liu et al., 2013b). In addition, 27 QTL were found for leaf and heading-related traits in a segregating population of Chinese cabbage (Ge et al., 2011). A segregating population of a cross between a Chinese cabbage with a turnip was used to identify loci related to morphological characteristics of the tap root (Lu et al., 2008). To investigate the genetic background of premature bolting under cold stress, a Chinese cabbage population was constructed based on crossing early and late bolting genotypes and QTL analysis identified 26 QTL (Wang et al., 2014c). Seven morphological traits were screened in a population derived from crossing a Chinese cabbage with a vegetable turnip and resulted in the detection of eight QTL (Kubo et al., 2010). A doubled haploid population of *B. oleracea* var. *capitata* served for detecting 13 QTL for heading-related traits (Lv et al., 2014). QTL analysis of a *B. oleracea* var. *italica* population, derived from a cross of a heat-sensitive and a heat-tolerant cultivar, identified AFLP markers correlated to floral development under heat stress (Lin et al., 2013). A high-density linkage map was established for a *B. oleracea* population segregating for carotenoid concentration in florets and three carotenoid QTL were found (Brown et al., 2014). Authors applied the *B. napus* SNP array and presented a 96% coverage of the *B. oleracea* genome. Localizing underlying factors affecting another major pigment, chlorophyll, in a Chinese cabbage population revealed five QTL for chlorophyll a and five QTL for chlorophyll b content (Ge et al., 2012). Vegetables with high nutrient use efficiency are also developed in order to reduce fertilizer application. Association mapping of *B. oleracea* accessions identified some QTL related to potassium concentration in shoot and those were tested using substitution lines (White et al., 2010). The gain of knowledge on the genetic localization of favorable traits is transferred to breeding new lines through marker-assisted selection. Accumulating three major loci for clubroot (*Plasmodiophora brassica*e) resistance genes resulted in the development of Chinese cabbage lines with strengthened resistance (Matsumoto et al., 2012).

**Table 1:** Published genomics analyses in the brassicaceous vegetables.

*Brassica* species	Population	Plant material	Reference
*B. oleracea*	Segregating population	Cross of contrasting geno-types	Camargo et al. (1997)
*B. oleracea*	Segregating population	Cross of contrasting genotypes	Sebastian et al. (2000)
*B. oleracea*	Segregating population	Cross of contrasting genotypes	Gao et al. (2007)
*B. oleracea*	Gene bank accessions	–	van Hintum et al. (2007)
*B. oleracea*	Doubled-haploid	Cross of contrasting genotypes	Iniguez-Luy et al. (2008)
*B. oleracea*	Gene bank accessions	DNA methylation	Salmon et al. (2008)
*B. oleracea*	TILLING	EMS mutagenesis	Himelblau et al. (2009)
*B. oleracea*	Gene bank accessions	–	White et al. (2010)
*B. oleracea*	Segregating population	Cross of contrasting genotypes	Brown et al. (2014)
*B. oleracea*		DNA methylation	Parkin et al. (2014)
*B. oleracea* var. *botrytis*	Segregating population	Cross of contrasting genotypes	Gu et al. (2008)
*B. oleracea* var. *capitata*	Gene bank accessions	–	Faltusova et al. (2011)
*B. oleracea* var. *capitata*	Doubled-haploid	Cross of contrasting genotypes	Wang et al. (2012)
*B. oleracea* var. *capitata*	Doubled-haploid	Cross of contrasting genotypes	Lv et al. (2014)
*B. oleracea* var. *italica*	Doubled-haploid	Cross of contrasting genotypes	Walley et al. (2012)
*B. oleracea* var. *italica*	Segregating population	Cross of contrasting genotypes	Lin et al. (2013)
*B. oleracea*, *B. rupestris*	Wild and cultivated populations, hybrid population		Maggioni et al. (2014)
*B. rapa*	Wild and cultivated populations	–	Takuno et al. (2007)
*B. rapa*	Segregating population	Cross of contrasting genotypes	Lu et al. (2008)
*B. rapa*	Segregating population	Cross of contrasting genotypes	Kubo et al. (2010)
*B. rapa*	TILLING	EMS mutagenesis	Stephenson et al. (2010)
*B. rapa*	Gene bank accessions	–	Zhao et al. (2010)
*B. rapa*	Segregating population, gene bank accessions	Cross of contrasting genotypes	Lou et al. (2011)
*B. rapa*	Segregating population	Cross of contrasting genotypes	Bagheri et al. (2012)
*B. rapa*	Hypermethylated population	DNA methylation	Amoah et al. (2012)
*B. rapa*	Segregating population	Cross of contrasting genotypes	Wang et al. (2014c)
*B. rapa* ssp. *pekinensis*	Segregating population	Cross of contrasting genotypes	Ge et al. (2011)
*B. rapa* ssp. *pekinensis*	Segregating population	Cross of contrasting genotypes	Ge et al. (2012)
*B. rapa* ssp. *pekinensis*	Marker-assisted selection	Gene pyramiding	Matsumoto et al. (2012)
*B. rapa* ssp. *pekinensis*	Doubled-haploid	Cross of contrasting genotypes	Liu et al. (2013b)

Advances in next generation sequencing technologies enabled surveying genotype-phenotype-relationships with the highest resolution to date (Wei et al., 2013; Varshney et al., 2014). A high-density linkage map was derived from the whole genome shotgun sequence of *B. oleracea* var. *capitata*, based on 1,227 genetic markers (Wang et al., 2012). The availability of *B. rapa* genome sequence aided in the generation of a linkage map for a recombinant inbred line descending from a vegetable leafy and an yellow sarson oilseed genotype of *B. rapa* (Bagheri et al., 2012). Recently, genomes of all three sequences *Brassicas* were compared to develop SSR markers and a total of 115,869, 185,662, and 356,522 primer pairs were designed from *B. rapa*, *B. oleracea,* and *B. napus*, respectively (Shi et al., 2014). EST markers were developed based on the sequenced transcriptomes of two cabbage lines susceptible or tolerant to black rot disease, demonstrating the feasibility especially for species without a reference genome sequence (Izzah et al., 2014).

One of the main strategies in reverse genetics is Targeting Induced Local Lesions IN Genomes (TILLING) and numerous applications are shown in functional genomics of model plants and crops (Kurowska et al., 2011; Chen et al., 2014). Random point mutations are generated by chemical mutagenesis and high-throughput screening for SNPs in the target gene isolates mutants with loss-of-function or gain-of-function phenotypes. Currently, two TILLING populations are established for brassicaceous vegetables, *B. oleracea*^1^ (Himelblau et al., 2009) and *B. rapa*^2^ (Stephenson et al., 2010) and both are freely accessible.

Next generation sequencing and AFLP mapping of methylation polymorphism allowed determining the DNA cytosine methylation status and, hence, enabled tackling the epigenome to understand gene expression variation (Niederhuth and Schmitz, 2014). The level of genome methylation was assessed in 30 *B. oleracea* populations and lines, and then related to phenotypic variability (Salmon et al., 2008). The *B. oleracea* genome was correlated to the leaf transcriptome and methylome and provided insights into polyplody events (Parkin et al., 2014). A chemically induced hypermethylated *B. rapa* population was developed recently and can serve for epiallele discovery (Amoah et al., 2012).

## Transcriptomics

Transcriptomics is based on the characterization and quantification of RNAs present in a given plant, organ, tissue, or cell. A particular attraction of assaying transcription is that it presages gene expression and so has the potential to provide a link between genotype and phenotype. Rapid developments in the relevant technologies now allow for the design of very large-scaled experiments which can in principle capture and enumerate every transcript present in a given biological sample (summarized in **Table 2**). The early transcriptomic platforms were built around the concept of immobilizing transcripts in an array, which was used to capture the mRNA content of a sample on the basis of nucleotide sequence complementarity. A more sophisticated form of microarray replaced transcripts with 20–70 nt long oligonucleotides designed to target known gene sequences (Knudsen, 2004). In both cases, the protocol requires bathing the array in a solution of labeled RNA extracted from the test sample. Hybridization of the probe at a given site on the array is detected by the fluorescence emitted by the labeled probe. Typically, two parallel experiments need to be run, in which one sample is derived from a plant exposed to a certain treatment while the other is derived from a plant which has not been exposed (control). Alternatively, genetic contrasts (such as wild type *vs.* a mutant, wild type *vs.* a transgenic) can replace the treatment contrast. Many of such experiments have been reported in a range of plant species (for example, Rabbani et al. (2003) in rice, Yu and Setter (2003) in maize, Lee and Yun (2006) in pepper). The quantity of such data collected from the brassicaceous vegetable species is relatively limited, partly perhaps because their full genome sequences have only very recently been acquired. The close phylogenetic relationship between *Brassica* spp. and *A. thaliana* has encouraged a number of attempts to use an *A. thaliana* microarray to analyze *Brassica* spp. transcriptomes. With the recent acquisition of *Brassica* spp. genome sequences, a number of *Brassica*-specific microarrays are finally becoming available – these include the *B. rapa* 24 K oligo microarray and the Agilent *Brassica* microarray, which is able to detect the transcription of >27,000 unigenes in a range of *Brassica* spp. The genetic closeness of *Brassica* spp. and *A. thaliana* has allowed large numbers of *Brassica* unigenes to be assigned a function, but nevertheless, substantial numbers appear to encode either *Brassica*-specific proteins or represent non-coding RNA (Trick et al., 2009).

**Table 2:** Published transcriptomic analyses in the brassicaceous vegetables.

*Brassica* species	Plant organ/developmental stage	Study objective	Methodology	Reference
*B. oleracea*	Flowers	Male sterility	Microarray	Kang et al. (2008)
*B. oleracea* var. *italica*	Seeds and sprouts	Glucosinolate metabolism	RNA-seq analysis	Gao et al. (2014)
*B. rapa*	Flowers	Self-incompatibility	Microarray	Kwun et al. (2004)
*B. rapa*	plants	Abiotic stress	Microarray	Lee et al. (2008)
*B. rapa*	Seedlings, roots, petioles, leaves, flowers	Comparative analysis	RNA-seq	Kim et al. (2012)
*B. rapa*	Seedlings, roots, leaves, petiole	Abiotic stress	RNA-Seq	Devisetty et al. (2014)
*B. rapa* ssp. *pekinensis*	Seedlings, root tips	Cold stress	Microarray	Yang et al. (2005)
*B. rapa* ssp.* pekinensis*	Leaves	Heat shock transcription factor	Comparative genomic analysis	Song et al. (2014)
*B. rapa* ssp. *pekinensis*	Seedlings	Plant-microbe interactions	RNA-seq	Sun (2014)
*B. rapa* ssp. *rapa*	Seedlings	Etiolation	RNA-seq	Zhou et al. (2014)

A cDNA microarray constructed from a Chinese cabbage (*B. rapa* ssp. *pekinensis*) pistil-specific cDNA library approach was used by Kwun et al. (2004) to investigate the effect of CO_2_ on self-incompatibility, and a similar strategy was followed by Yang et al. (2005) to identify genes up-regulated by low temperature stress. Meanwhile Lee et al. (2008) synthesized an oligo-based microarray from the sequences of 24,000 *B. rapa* unigenes to characterize the response to low temperature, salinity and drought. Based on the identification of candidate genes using a 2 × 104 k *Brassica* microarray to compare the gene expression of untreated and methyl jasmonate treated pak choi sprouts, Wiesner et al. (2013a, 2014) were able to identify the homologues genes involved in the synthesis of 1-methoxyindole-3-ylmethyl glucosinolate.

The major alternative to microarray technology relies on amplification rather than on hybridization. The most commonly used method, termed RNA-seq, is designed to generate a *de novo* assembly of the transcriptome. This has largely replaced the earlier approach called “serial analysis of gene expression” (SAGE). In general, a population of RNA (total or fractionated) is converted to a library of cDNA fragments with adaptors attached to one or both ends. Each molecule, with or without amplification, is then sequenced in a high-throughput manner to obtain short sequences from one end (single-end sequencing) or both ends (pair-end sequencing). The reads are typically 300–400 bp, depending on the DNA-sequencing technology used (Wang et al., 2009). In principle, any high-throughput sequencing technology can be used for RNA-Seq. The data generated from RNA-Seq experiments are in the form of an absolute frequency of each transcript identified, which obviates the need for using a reference sequence. For this reason, it can provide a greater level of insight and accuracy than microarray analysis can (Marioni et al., 2008; T’Hoen et al., 2008). In broccoli, the RNA-Seq approach has been taken to describe the transcription of genes involved in glucosinolate metabolism (Gao et al., 2014). Of the nearly 20 k unigenes recovered, more than 2,500 appeared to be differentially transcribed in a contrast between the seed and the seedling, and a large proportion of these were putative transcription factors. Curiously, the up-regulation of candidate glucosinolate synthesis genes was negatively correlated with the glucosinolate content of the germinating seedling; a possible explanation offered by the authors was that the high transcript abundance of an *TGG2* orthologue (encoding a myrosinase) had the effect of degrading aliphatic glucosinolates (Gao et al., 2014).

Small RNAs, which range in length from 19 to 25 nt (most are either 21 nt or 24 nt long), are ubiquitous in eucaryotic cells. Extensive transcriptome sequencing has revealed the presence of a large number of such RNAs, which cannot be translated into a protein, but rather act as an important class of regulators, especially in the context of plant development, signal transduction, metabolism and the response to biotic and abiotic stress (Ellendorff et al., 2009; Sun, 2012). The abundance of a particular species of a class of small RNAs, referred to as microRNAs (miRNAs), varies from plant species to plant species, from developmental stage to developmental stage and from tissue to tissue (Carrington and Ambros, 2003; Bartel, 2004; Kenan-Eichler et al., 2011). The transcriptional response of Chinese cabbage to infection by *Erwinia carotovora* ssp. *carotovora* included a marked alteration in the abundance of certain small RNAs (Sun, 2014). In turnip (*B. rapa* ssp. *rapa*), it appeared that various miRNAs are involved in the regulation of plant growth, development and differentiation in the absence of light (Zhou et al., 2014). A number of *Brassica* species are polyploids, the genomes of which have been markedly altered by the polyploidization event (Kenan-Eichler et al., 2011). Some of these changes have been associated with the activity of small RNAs. Kim et al. (2012) identified 412 distinct miRNAs in *B. rapa*, of which 216 were novel. The same study identified 29 novel miRNAs which were only found in the flower.

## Proteomics

The acquisition of the complete genome sequence of a growing number of plant species^3^ along with their transcriptomes continues apace. However, much of the physiology of the cell is determined by gene products (particularly, but not exclusively, proteins) rather than by nucleic acid. A proteomic analysis seeks to characterize the full protein complement present in a particular organism, organ, tissue, or cell. Initial attempts to derive this were based on two dimensional gel electrophoresis (2-DE), as described by O’Farrell (1975). Besides 2-DE, current proteomics platforms exploit more versatile LC (liquid chromatography)-based methods (Rabilloud et al., 2010; Matros et al., 2011), coupled with mass spectrometry (MS). Complicating the analysis, and unlike the nucleic acids, protein molecules are subject to a range of functional modification, such as phosphorylation, glycosylation, and acetylation (Cox and Mann, 2011). Nevertheless, proteomic approaches have been successfully applied to a number of plant species to study various developmental processes and environmental adaptation (see reviews by Barkla et al., 2013; Vanderschuren et al., 2013; Zhuang et al., 2014). Kehr and Buhtz (2011) have provided a summary of the literature regarding proteomic analyses in *Brassica* spp., a list dominated by experiments based on oilseed rape. In the following, we give emphasis to reports on how proteomic techniques are applied to catalog and characterize special, developmental-driven or environmentally induced alterations in the proteome of *brassicaceous* vegetables (summarized in **Table 3**).

**Table 3:** Published proteomic analyses in the brassicaceous vegetables.

*Brassica* species	Plant organ/developmental stage	Study objective	Methodology	Reference
Members of *Brassicaceae* family	Etiolated leaves of seedlings	Genotypic variation	2-DE	Marquès et al. (2001)
*B. oleracea* var. *capitata*, *B. rapa* var. *pekinensis*, synthesized *B. napus*	Seeds	Genotypic variation	2-DE and MS/MS	Hossain et al. (2014)
*B. oleracea*	Xylem saps	Mapping	1-DE and MS/MS	Buhtz et al. (2004)
*B. oleracea*	Vacuoles	Mapping	MS/MS	Schmidt et al. (2007)
*B. oleracea*	Xylem saps	Mapping, *N*-glycosylation	MS/MS	Ligat et al. (2011)
*B. oleracea*	Phloem tissues of stem	Mapping	MS/MS	Anstead et al. (2013)
*B. oleracea* var. *alboglabra*	Leaves, stems	Genotypic variation	2-DE	Albertin et al. (2005)
*B. oleracea* var. *alboglabra*	Leaves	Genotypic variation	2-DE and MS	Kong et al. (2010)
*B. oleracea* var. *botrytis italica*	Leaves, stems	Genotypic variation	2-DE	Albertin et al. (2005)
*B. oleracea* var. *botrytis*	Mitochondria	Mapping	2-DE and MS	Pawlowski et al. (2005)
*B. oleracea* var*. capitata*	Floral head	Effect of transgene	2-DE and MS/MS	Liu et al. (2011)
*B. oleracea* L. var *capitata*	Leaves	Cropping systems	2-DE and MS/MS	Nawrocki et al. (2011)
*B. oleracea* var*. capitata*	Stigma	Self-incompatibility	2-DE	Zeng et al. (2012)
*B. oleracea* var*. capitata*	Floral head	Effect of transgene	2-DE and MS/MS	Liu et al. (2013a)
*B. oleracea* var*. capitata*	Seeds	Genotypic variation	2-DE and MS/MS	Hossain et al. (2014)
*B. oleracea* var*. capitata*	Floral heads	Effect of transgene	2-DE and MS/MS	Liu et al. (2014a)
*B. oleracea* var*. italica*	Xylem sap	Salinity	2-DE and MS/MS	Fernandez-Garcia et al. (2011)
*B. oleracea* var. *italica*	Floral heads	Sodium selenate nutrition	2-DE and MS/MS	Sepúlveda et al. (2013)
*B. rapa cvs.* Natsumaki, Takamaru	Roots	Pathogen interaction	2-DE	Kaido et al. (2007)
*B. rapa*	Leaves	Genotypic variation	2-DE and MS	Kong et al. (2010)
*B. rapa* ssp. *chinensis*	Leaves	Effect of di-*n*-butyl phtalate	2-DE and MS/MS	Liao et al. (2006)
*B. rapa* var. *chinensis*	Leaves	Effect of di-*n*-butyl phtalate	2-DE and MS/MS	Liao et al. (2009)
*B. rapa* ssp. *chinensis*	Leaves	Mapping	2-DE	Alam et al. (2013)
*B. rapa* ssp. *chinensis*	Leaves	Pathogen interaction	2-DE and MS/MS	Sun et al. (2014)
*B. rapa* ssp. *chinensis*	Pistil	Self-incompatibility	2-DE and MS/MS	Wang et al. (2014a)
*B. rapa* ssp. *chinensis*	Leaves and roots	Nitrogen nutrition	2-DE and MS	Zhuang et al. (2014)
*B. rapa* ssp. *pekinensis*	Seedlings	Effect of atrazine	2-DE and MS	Li et al. (2008)
*B. rapa* var. *pekinensis*	Seeds	Genotypic variation	2-DE and MS/MS	Hossain et al. (2014)

Using a conventional 2-DE approach, Alam et al. (2013) succeeded in detecting about 1,300 distinct low abundance leaf proteins in Chinese cabbage. A characterization of the proteomic content of the xylem sap of broccoli and oilseed rape (and some non- brassicaceous) undertaken by Buhtz et al. (2004) showed little evidence of any species specificity, while De Bernonville et al. (2014) were able to define the proteomic impact in the xylem sap induced by infection of *B. oleracea* with the pathogen *Xanthomonas campestris* pv. *campestris*. Ligat et al. (2011) expanded the *B. oleracea* data set by subjecting the xylem sap proteome to LC-MS/MS and the *N*-glycoproteome by prior enrichment with concanavalin A affinity chromatography and LC-MS/MS. Most of the ∼200 proteins identified proved to be involved in cell wall-related carbohydrate metabolism, although a number of oxido-reductases and proteases were also revealed. A proteomic analysis of broccoli tissue enriched with phloem allowed the identification of 379 proteins, some of which were structural and others associated with the biotic and/or abiotic stress response (Anstead et al., 2013). In cauliflower, an analysis of isolated tonoplast membranes revealed 102 tonoplast integral and 214 peripheral proteins (Schmidt et al., 2007). A further study in this species was able to show that its mitochondrial proteome was highly similar to that of *A. thaliana*, and included at least 51 proteins involved in the generation of ATP, protein folding and protein transport (Pawlowski et al., 2005).

The host-pathogen interaction is important in brassicaceous vegetables, as in all crops. A root proteomic comparison between a pair of turnip cultivars showing a contrasting reaction to the causal agent of clubroot disease was able to demonstrate the presence of certain proteins in the resistant cultivar which were absent from the profile of the sensitive one (Kaido et al., 2007). Sun et al. (2014) tracked the proteomic response of Chinese cabbage resulting from infection with downy mildew (*Hyaloperonospora parasitica*), and were able to identify some 91 proteins which altered in their abundance; some of these were involved in ethylene signaling. When the abundances of specific transcripts and their gene products were compared, a correlation could be established for only about half of the 33 genes investigated; a finding which was interpreted as implying that many of the early events in the host’s resistance response involved post-translational modification.

Abiotic stress is a further major production constraint. Analysis of the effect of the photosystem II inhibitor atrazine on the chloroplast proteome of Chinese cabbage has identified at least nine proteins as being differentially expressed; some of these were related to isoprene or protein synthesis (Li et al., 2008). Investigations of the impact of the industrial soil pollutant phthalate ester on either pak choi (Liao et al., 2006) or Chinese cabbage (Liao et al., 2009) have shown that the pollutant was responsible for a different set of up- or down-regulated proteins, even though the two species are so closely related to one another. Among the effects of salinity on broccoli are the down-regulation of several proteins involved in cell wall metabolism and the up-regulation of known plant defense-related proteins (Fernandez-Garcia et al., 2011). A third common source of abiotic stress is that imposed by nutrient supply. Wang et al. (2014b) have characterized the response of hydroponically grown pak choi to the supply of nitrogen in different forms. The plants responded to glycine supply by altering the expression of proteins involved in protein processing, amino acid metabolism and redox homeostasis. A similar experiment involving broccoli investigated the effect of supplying the plants with a non-limiting concentration of the essential trace element selenium (Sepúlveda et al., 2013), resulting in an enhanced accumulation in the inflorescence of a number of proteins associated with the general stress response, while that of pathogen defense-related proteins was reduced. The effect of an organic farming regime on the cabbage proteome has shown that the cropping system had a measurable effect on the accumulation of proteins involved in glycolysis, the Krebs cycle, amino acid metabolism and various detoxification processes (Nawrocki et al., 2011).

A plants protein composition is a key determinant of a species’ and a cultivar’s identity, and differences in specific plant features are better reflected by their proteome than by either their genome or transcriptome. Thus proteome variation has been suggested as providing a viable basis for the design of molecular markers able to accelerate breeding (Witzel et al., 2011). Marquès et al. (2001) made comparisons between the 2-DE profiles of various *Brassica* species in an attempt to establish the nature of the genetic relationship between them. PCR-based markers for the genomes A (*B. rapa*) and B (*B. oleracea*) were obtained by Kong et al. (2010) by aligning the proteomic profiles of young leaves. From some of those differentially expressed proteins, PCR-based markers were developed successfully. The effect of the polyploidization process on the proteome was assessed by Albertin et al. (2005), who made comparisons between the leaf and stem proteomes of haploid, diploid and tetraploid sprouting broccoli, and Chinese kale. The analysis concluded that the two different tissues were responsible for 40% of the proteomic variation observed, with only 10% ascribed to ploidy level differences. With the aim to identify proteins responsible for an improved vernalisation trait, the seed proteome of a somatic hybrid line was compared with those of its parent lines *B. oleracea* (cabbage) and *B. rapa* (Chinese cabbage) that showed low or high seed production during warm winter conditions, respectively (Hossain et al., 2014).

Understanding the basis of self-incompatibility has been a long-standing problem in the *Brassica* genus. A proteomic approach addressing this issue in Chinese cabbage was taken by Wang et al. (2014a), who analyzed the protein complement of the pistils from self-incompatible and compatible plants; in some cases, protein abundance was consistent with that of the relevant transcript, particularly with respect to those involved in energy metabolism, protein synthesis and the defense against stress. Recently, for a more cell-type specific proteomics approach related to self-incompatibility, a protocol for the analysis of cabbage stigma proteins was established (Zeng et al., 2012).

The market value of vegetables is greatly affected by their visual appearance and their post-harvest shelf life. As part of a transgenic-based attempt to prolong the marketability of broccoli, 2-DE was used to compare the proteomes of the inflorescences of wild type and transgenic lines produced to express delayed senescence (Liu et al., 2011). The conclusion was that a number of stress response related proteins and chaperones accumulated in the transgenic lines, while the build-up of 1-aminocyclopropane-1-carboxylate oxidase was only noted in the wild type. When the effect of exogenously supplying cytokinin (N^6^-benzylaminopurine) was studied at the proteomic level, Liu et al. (2013a) detected only a minor degree of overlap with the transgenic contrast, an observation which was taken to suggest that the molecular basis of the delayed post-harvest senescence induced by exogenous cytokinin differed from that induced by endogenous cytokinin. Of the up-regulated stress response related proteins detected in the transgenic material, 17 proved to be heat-stable following cooking and were therefore considered to represent potential allergens (Liu et al., 2014a).

## Metabolomics

Metabolomics, defined as the systematic study of the by-products of cellular processes, is an ambitious field, as the number of chemically distinct molecules involved in a typical plant sample is estimated to be at least 100,000 (Wink, 1988), each varying with respect to their abundance (De Vos et al., 2007). A number of these compounds are of physiological importance and/or represent dietary sources of antioxidants and other health enhancing compounds; most importantly with respect to vegetable crops, they make a major contribution to product color and flavor. Often these latter quality aspects rely not on individual compounds, but rather on specific mixtures (Ferguson and Schlothauer, 2012). The state-of-the-art analytical platforms employed for metabolomic analyses are a sophisticated combination of nuclear magnetic resonance spectroscopy (NMR), MS, gas chromatography (GC), LC, and capillary electrophoresis (CE). Changes in the primary and secondary metabolite pool have been characterized using a GC-MS approach (Hummel et al., 2013), while CE-MS is more suitable for characterizing the products associated with the central metabolism (Yang et al., 2012). NMR is appropriate for the targeting of phenolic compounds, carbohydrates, organic acids and amino acids, and of particular interest in the context of brassicaceous vegetables, glucosinolates (**Table 4**). Electrospray ionization (ESI), conducted either in positive (resulting in protonated species) or in negative (deprotonated species) mode is used for the analysis of semi-polar metabolites. Together, these technologies can identify and quantify a wide range of primary and secondary metabolites. For LC-MS and NMR applications, samples are typically extracted in alcohol and not derivatized. In contrast, GC-MS separations require a derivatization step (Kopka et al., 2005). Particularly high mass resolution can be achieved by the use of time-of-flight (TOF)-MS devices, and additional information can be obtained from the MS/MS fragmentation patterns and UV-VIS spectra. Recent software developments have improved the capacity to recognize different metabolites. Profiles based on mass, retention time and signal amplitude provide the data required for filtering biomarkers. Typically a data processing pipeline can be divided into two steps: data processing (filtering, feature detection, alignment, and normalization) and data analysis (algorithm selection, training, evaluation, and model examination; further explanation see Boccard et al., 2010).

**Table 4:** Published non-targeted metabolomic analyses in the brassicaceous vegetables.

*Brassica* species	Plant organ/developmental stage	Study objective	Methodology	Compounds	Reference
*B. oleracea* collection	Leaves	Factor identification for thermal degradation of glucosinolates	HPLC-MS LC-MS	Semi-polar	Hennig et al. (2014)
*B. oleracea* var. *botrytis* collection	Inflorescences	Genotypic variation of colored cultivars	GC-TOF-MS	Primary and secondary compounds*	Park et al. (2013)
*B. oleracea* var. *capitata*	Leaves	Discrimination of conventional and organic farming	LC-MS	Semi-polar	Mie et al. (2014)
*B. oleracea* var. *capitata*	Leaves	Genotypic variation	LC-MS	Phenolic compounds	Park et al. (2014)
*B. oleracea* var. *italic*	Inflorescences	Genotypic variation, selenium treatment, crop management	LC-MS	Phenolic compounds	Luthria et al. (2008)
*B. rapa* collection	Leaves	Response to pre-harvest bacterial contamination	^1^H NMR	Polar	Jahangir et al. (2008b)
*B. rapa* collection	Leaves	Genotypic variation	^1^H NMR	Polar	Abdel-Farid et al. (2007)
*B. rapa* collection	Leaves	Effect of metal-ion treatment	^1^H NMR	Polar	Jahangir et al. (2008a)
*B. rapa* collection	Leaves	Response to pre-harvest fungal infection	^1^H NMR	Polar	Abdel-Farid et al. (2009)
*B. rapa collection*	Leaves	Genotypic variation	LC-MS	Semi-polar	Del Carpio et al. (2011)
*B. rapa* var. *pekinensis*	Leaves	Discrimination by geographical areas	^1^H NMR	Polar	Kim et al. (2013a)
*B. rapa* var. *rapa*, *Raphanus sativa*	Leaves	Ontogenetic variation	NMR, HPLC	Primary and secondary compounds*	Muhammad et al. (2014)

*Includes both a non-targeted and a targeted approach.

Nevertheless, metabolite identification in non-targeted approaches in the absence of authentic reference compounds remains difficult. Various platforms and spectral databases are available online^4^.

The metabolome of *Brassica* spp. as that of all plants, is influenced by both genotype (Luthria et al., 2008) and developmental stage (Abdel-Farid et al., 2007), as well as by the plant’s growing environment (Hall, 2006; Weimer and Slupsky, 2013). Metabolomic analyses in the brassicaceous vegetables have included both non-targeted (**Table 4**) and targeted (**Table 5**) approaches (Cevallos-Cevallos et al., 2009). The former have concern a range of both secondary (polyphenols and carotenoids) and primary metabolites (Hall, 2006), while the latter focused heavily on the identification and quantification of glucosinolates (Ramautar et al., 2006). The glucosinolates are a unique component of the brassicaceous vegetable metabolome (Verkerk et al., 2009). These compounds contain a sulfur-linked β-D-glucopyranose moiety and an amino acid-derived side chain, and fall into three definable classes: the aliphatic, indole and aromatic glucosinolates. They are structurally highly diverse, and within the *Brassicaceae* are mostly dominated by the aliphatic glucosinolates (Halkier and Gershenzon, 2006). Subsequently, aliphatic glucosinolates can be subdivided into straight- or branched-chain alk(en)yl glucosinolates with or without a hydroxy group and into a large group of those that contain an additional sulfur atom with various oxidation levels in the side chain (see for detailed structural overview, Hanschen et al., 2014). The glucosinolates are not known to be bioactive, but their hydrolysis products provide deterrence against feeding (Textor and Gershenzon, 2009), and their presence in the human diet has been associated with both health benefits (Vig et al., 2009) and taste (Beck et al., 2014).

**Table 5:** Published targeted metabolomic analyses in the brassicaceous vegetables, featuring glucosinolates and their break-down products.

*Brassica* species	Plant organ/Developmental stage	Study objective	Methodology	Reference
*B. carinata*	Leaves	(Onto)genetic variation, effect of water supply	HPLC-MS	Schreiner et al. (2009)
*B. oleracea*	Leaves	Genotypic variation	LC-MS	Rochfort et al. (2008)
*B. oleracea* var. *italica*	Inflorescences	Effect of nitrogen and sulfur supply	HPLC-MS	Schonhof et al. (2007a)
*B. oleracea* var. *italica*	Inflorescences	Effect of CO_2_	HPLC-MS	Schonhof et al. (2007b)
*B. oleracea* var. *italica*	Sprouts	Effect of water supply and aphid treatment	HPLC-MS	Khan et al. (2011)
*B. oleracea* var. *italica*	Sprouts	Effect of UV-B	HPLC-MS	Mewis et al. (2012)
*B. rapa*	Roots	Effect of nitrogen and sulfur supply	HPLC-MS	Shumin et al. (2007)
*B. rapa*	Leaves	Effect of fungal infection	NMR	Abdel-Farid et al. (2010)
*B. rapa*	Leaves	Effect of fertilization	GC-MS	Okazaki et al. (2012)
*B. rapa*	Roots	Genotypic variation	LC-PDA-QTOF-MS	Lee etal. (2013)
*B. rapa* ssp. *chinensis*	Leaves, sprouts	Effect of methyl jasmonate	HPLC-MS	Wiesner et al. (2013a,b)
*B. rapa* ssp*. pekinensis*	Leaves, seeds	Organ differentiation	LC-ESI-MS, LC-UV	Hong et al. (2011)
*B. rapa* ssp. *rapa*	Leaves, roots, root exsudates	Effect of methyl jasmonate, salicylic acid	HPLC-MS	Schreiner et al. (2011)

Gas chromatography/TOF-MS has been used to characterize the phytochemical diversity of the florets in cauliflower cultivars varying in inflorescence pigmentation (Park et al., 2013). A ^1^H-NMR platform proved informative for discriminating between a set of Chinese cabbage cultivars from China and Korea: differences with respect to both primary and secondary metabolites were uncovered, although insufficient attention has yet to be given to the effect of the growing environment (Kim et al., 2013a). A non-targeted strategy was chosen by Mie et al. (2014) to reveal the impact of organic farming on the metabolome of cabbage, and led to the conclusion that this approach has potential for the authentication of organic products. The exposure of *B. carinata* seedlings to lithium ions induced a marked effect on the plant’s lipid and phenolic content: sinapic acid esters and chloroplast lipids were replaced by benzoate derivatives, resveratrol and oxylipins (Li et al., 2009). When GC/TOF-MS was used to discriminate between cabbage cultivars varying with respect to their resistance to feeding by diamondback moth (*Plutella xylostella*) caterpillars, Kim et al. (2013b) were able to show that the levels of glycolic acid, quinic acid, inositol, fumaric acid, glyceric acid, trehalose, shikimic acid, and aspartic acid were all very different.

Non-targeted metabolic approaches have also been applied to the study of processing quality in brassicaceous vegetables. Changes in the content of certain flavonols have been associated with the thermal degradation of glucosinolates in *B. oleracea* (Hennig et al., 2014). Several analytical techniques have been used to assess the impact of industrial processing on the phytochemical composition of vegetable purées. In broccoli, most of the metabolites present in the purée were degraded by heating, including various health-related and flavor compounds, vitamins, carotenoids, flavonoids, glucosinolates, and oxylipins (Lopez-Sanchez et al., 2015). Post-harvest storage temperature also has a profound effect on the metabolome. For example, in radish *(Raphanus sativus*), the content of phenylpropanoids, flavonoids, and glucosinolates decreased with storage time (Jahangir, 2010).

## Microbiomics

Microbiomics refers to the application of ‘omics technologies to the microbial community associated with the plant spermosphere, endosphere, rhizosphere, and phyllosphere. The plant microbiome has attracted the focus of several researchers in recent years (reviewed in Vorholt, 2012; Bakker et al., 2013; Bulgarelli et al., 2013), as it has an intimate interaction with the plant, affecting both its metabolism and physiology. Studies of the plant microbiome have relied to date either on the *in vitro* culture of the relevant micro-organisms or on the recognition of species identity from PCR amplicons derived from variable regions of their genome (such as the 16S rDNA locus) followed by sequencing, microarray analysis or various electrophoretic procedures (Rastogi et al., 2013; Turner et al., 2013; Muller and Ruppel, 2014). Metaproteomics approaches can complement such data sets to provide functional information relevant to the microbiome (Knief et al., 2012). As many vegetable are consumed as a fresh product, their associated microbiome will enter the human digestive system. Exemplarily, the lettuce (Hunter et al., 2010; Rastogi et al., 2012; Jackson et al., 2013; Williams and Marco, 2014) and spinach (Leff and Fierer, 2013; Lopez-Velasco et al., 2013) microbiomes are dependent on the host genotype, the cultivation conditions and the tissue sampled. The impact of commensal microbial diversity on crop disease has been investigated in a range of oilseed rape cultivars infected with the fungal pathogen *Verticillium longisporum* (Graner et al., 2003). Bacterial strains isolated from tolerant or susceptible plant cultivars showed biocontrol activity against the fungus and imply a possible application in crop protection. Meanwhile De Campos et al. (2013) used next generation sequencing to show how the components of the oilseed rape microbiome depended on the developmental stage of the plant. Links et al. (2014) demonstrated that the seeds of *B. juncea, B. napus,* and *B. rapa*, were associated with a core seed microbial population of geographically and ecologically different crops. The structure of the *Brassica* species -specific microbiome has been shown to be sensitive to the composition of the host’s secondary metabolites (Ruppel et al., 2008). Hunter et al. (2014) established a close relationship between the composition of the root exudate and the structure of the rhizosphere microbiome. Certain microbial strains had a significant impact on the capacity of the rhizosphere to solubilize phosphorus (Schilling et al., 1998; Deubel et al., 2000). Determining the functional interaction between the microbiome’s composition and plant metabolic activities is still at an early stage of development (Bakker et al., 2013).

## Concluding Remarks

**FIGURE 2:**
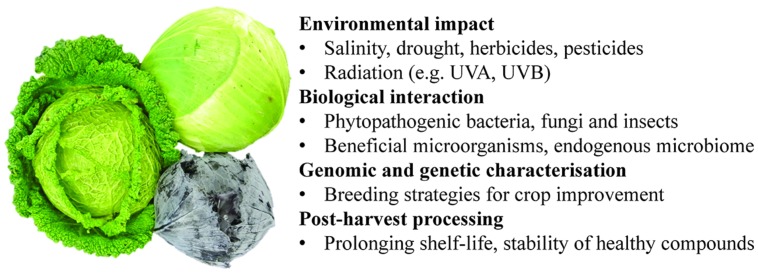
**Potential applications of ‘omics technologies for brassicaceous vegetables improvement**.

Many refinements in all the ‘omics technologies can be expected to come on-stream over the next few years, and these will provide exciting new avenues of research in the brassicaceous vegetables. Some of the topics likely to feature in the near future are flux analysis, the identification of informative breeding markers for the selection of beneficial bioactives and enhanced responses to both abiotic and biotic stress (**Figure 2**). The increasing volume of transcriptomic data acquired continues to provide a wealth of information relevant to obtaining a detailed picture of key regulatory mechanisms and pathways active in the plant. Improving the capacity to identify the components of the proteome is a fast moving area of technology development. So far, proteomic analysis in the brassicaceous vegetables has relied almost exclusively on 2-DE separation, even though the more sophisticated chromatography and MS platforms have been quite widely used in other (particularly model) plant species; these technologies are of particular value in illuminating post-translational modifications. The acquisition of full genome sequences for some (or all) of the brassicaceous vegetables can only aid in facilitating proteomic analysis in this groups of crop plants. Gaining some control over the composition of metabolites will surely provide a powerful means of varietal improvement and the optimization of post-harvest technology.

The marriage of ‘omics technology and plant breeding should trigger a major efficiency breakthrough in crop improvement, and also generate novel opportunities in the fields of nutrigenomics (systems approach to understand the relationship between diet and health) and microbiomics. Although substantial progress has already been made both in the data acquisition and its interpretation, many technical and scientific challenges remain before the level of understanding of the workings of a complex biological system such as a plant rises above the current state. Global information of different hierarchies (genomics, transcriptomics, proteomics, and metabolomics) need to be integrated using mathematical and statistical methods to refine existing knowledge and make new discoveries.

There are successful studies were systems perspectives revealed correlations that had not been suggested by classical approaches in *Arabidopsis,* (Mochida and Shinozaki, 2011), tomato (Enfissi et al., 2010), maize (Casati et al., 2011), and others. Elucidating principles that govern relationships between biological instances will help to improve plant functions also in brassicaceous vegetables; i.e., stress-resistant plants and high- yield cultivars.

## Conflict of Interest Statement

The authors declare that the research was conducted in the absence of any commercial or financial relationships that could be construed as a potential conflict of interest.

## Footnotes

1 http://www3.botany.ubc.ca/can-till

2 http://revgenuk.jic.ac.uk

3 phytozome.jgi.doe.gov/

4 www.metabolomicsociety.org
